# Characterization and Application of Synergistically Degraded Chitosan in Aquafeeds to Promote Immunity, Antioxidative Status, and Disease Resistance in Nile Tilapia (*Oreochromis niloticus*)

**DOI:** 10.3390/polym17152101

**Published:** 2025-07-31

**Authors:** Thitirat Rattanawongwiboon, Natthapong Paankhao, Wararut Buncharoen, Nantipa Pansawat, Benchawan Kumwan, Pakapon Meachasompop, Phunsin Kantha, Tanavan Pansiri, Theeranan Tangthong, Sakchai Laksee, Suwinai Paankhao, Kittipong Promsee, Mongkhon Jaroenkittaweewong, Pattra Lertsarawut, Prapansak Srisapoome, Kasinee Hemvichian, Anurak Uchuwittayakul

**Affiliations:** 1 Thailand Institute of Nuclear Technology (Public Organization), Nakorn Nayok 26120, Thailand; thitirat@tint.or.th (T.R.); tanavan.pan@gmail.com (T.P.); theeranan@tint.or.th (T.T.); sakchai@tint.or.th (S.L.); pattra@tint.or.th (P.L.); 2 Kamphaeng Saen Fisheries Research Station, Faculty of Fisheries, Kasetsart University, Kamphaeng Saen Campus, Nakhon Pathom 73140, Thailand; ffisnpp@ku.ac.th (N.P.); ongku52@gmail.com (S.P.); pomsri03@gmail.com (K.P.); mongkhon3112@gmail.com (M.J.); 3 Department of Biology, Faculty of Science, Chiang Mai University, Chiang Mai 50200, Thailand; wararut.bun@cmu.ac.th; 4 Department of Fishery Products, Faculty of Fisheries, Kasetsart University, Bangkok 10900, Thailand; ffisntp@ku.ac.th; 5 Department of Aquaculture, Faculty of Fisheries, Kasetsart University, Bangkok 10900, Thailand; benchawan.kumw@ku.th (B.K.); pakapon.meac@ku.th (P.M.); phunsin.k@ku.th (P.K.); ffispssp@ku.ac.th (P.S.); 6 Laboratory of Aquatic Animal Health Management, Department of Aquaculture, Faculty of Fisheries, Kasetsart University, Bangkok 10900, Thailand; 7 Center of Excellence in Aquatic Animal Health Management, Faculty of Fisheries, Kasetsart University, Bangkok 10900, Thailand

**Keywords:** chitosan, radiation, hydrogen peroxide, dietary supplement, Nile tilapia, low-molecular-weight chitosan, antioxidant response, *Edwardsiella tarda*

## Abstract

This study investigated the immunonutritional potential of high-molecular-weight (Mw~85 kDa), non-degraded chitosan (NCS) and gamma-radiation-degraded, low-molecular-weight chitosan (RCS) incorporated into aquafeeds for Nile tilapia (*Oreochromis niloticus*). RCS was produced by γ-irradiation (10 kGy) in the presence of 0.25% (*w*/*v*) H_2_O_2_, yielding low-viscosity, colloidally stable nanoparticles with Mw ranging from 10 to 13 kDa. Five diets were formulated: a control, NCS at 0.50%, and RCS at 0.025%, 0.050%, and 0.075%. No adverse effects on growth were observed, confirming safety. Immune gene expression (e.g., *ifng1*, *nfκb*, *tnf*), antioxidant markers (e.g., reduced MDA, increased GSH and GR), and nonspecific humoral responses (lysozyme, IgM, and bactericidal activity) were significantly enhanced in the NCS-0.50, RCS-0.050, and RCS-0.075 groups. Notably, these benefits were achieved with RCS at 10-fold lower concentrations than NCS. Following challenge with *Edwardsiella tarda*, fish fed RCS-0.050 and RCS-0.075 diets exhibited the highest survival rates and relative percent survival, highlighting robust activation of innate and adaptive immunity alongside redox defense. These results support the use of low-Mw RCS as a biologically potent, cost-effective alternative to traditional high-Mw chitosan in functional aquafeeds. RCS-0.050 and RCS-0.075 show strong potential as immunonutritional agents to enhance fish health and disease resistance in aquaculture.

## 1. Introduction

The global human population continues to grow at an unprecedented rate, driving a parallel increase in demand for high-quality, protein-rich food sources. Aquaculture has emerged as a critical sector to meet this demand, with Nile tilapia (*Oreochromis niloticus*) ranking among the most extensively farmed species worldwide due to its rapid growth, broad environmental tolerance, and favorable nutritional profile [1,2]. In Thailand, tilapia culture represents a cornerstone of rural livelihoods and domestic food security [3]. However, the intensification of production often exacerbates disease outbreaks, most notably edwardsiellosis caused by *Edwardsiella tarda*, which can lead to high mortality rates, reduced feed intake, and significant economic losses, particularly during summer or in rising temperature conditions [4]. Concurrently, oxidative stress resulting from intensive culture or suboptimal conditions further compromises fish health [5]. To sustain productivity, there is an urgent need for feed strategies that not only supply essential nutrients but also bolster fish immunity and resilience against pathogens such as *E. tarda*.

Chitosan, a deacetylated derivative of chitin which is the second most abundant natural polymer, offers a promising avenue for such functional feeds. It is biodegradable, biocompatible, non-toxic, and sourced economically from marine crustacean waste, of which Thailand is a leading global exporter [6,7]. The physicochemical and biological activities of chitosan, including antimicrobial, antioxidant, and immunomodulatory effects, are highly dependent on its molecular weight (MW) and distribution. High-MW chitosan (average molecular weight, Mw ≈ 300–400 kDa; 200,000–500,000 Da) exhibits excellent film-forming and viscosity properties but limited bioactivity [8], whereas low-MW chitosan (Mw ≈ 50–80 kDa; 10,000–100,000 Da) demonstrates superior biological functions such as antimicrobial, antioxidant, antitumor, and immune-enhancing properties [8,9].

Conventional degradation methods (thermal, enzymatic, ultrasonic, or ionizing radiation) each have limitations in efficiency, environmental impact, or functional-group preservation. Radiation degradation, in contrast, can effectively reduce chain length without the need for chemical initiators, maintaining the integrity of chitosan’s active amino and hydroxyl groups [9,10]. Recent studies have begun to explore chitosan as a dietary supplement in aquaculture, as the dietary inclusion of chitosan has been shown to improve growth performance, inhibit intestinal pathogens, and enhance innate immunity in various fish species [11,12,13]. Despite these promising findings, studies specifically addressing the combined effect of synergistic degradation methods (e.g., radiation combination with hydrogen peroxide) and their application in Thai tilapia farming remain limited.

To address this knowledge gap, the present study aims to (1) characterize low-MW chitosan produced via synergistic degradation by gamma irradiation and hydrogen peroxide, leveraging the high reactivity of hydroxyl radicals for rapid depolymerization, and (2) evaluate its efficacy as a functional feed additive to promote immunity, antioxidative status, and disease resistance in Nile tilapia under laboratory conditions. We employed gel permeation chromatography (GPC), viscometry, and dynamic light scattering (DLS) to assess molecular-weight distribution, viscosity, and particle size, respectively, and conducted in vitro antimicrobial assays to confirm bioactivity. Subsequently, chitosan-coated pellets were performed and fed to tilapia, with growth performance, feed efficiency, immune responses, antioxidant enzyme activities, and pathogen challenge survival recorded. By integrating advanced chitosan bioprocessing with comprehensive in vivo evaluation, this work seeks to deliver a sustainable, value-added strategy for enhancing tilapia health and productivity in Thailand’s aquaculture industry.

## 2. Materials and Methods

### 2.1. Development and Characterization of Synergistically Degraded Chitosan

#### 2.1.1. Materials

Chitosan (degree of deacetylation ≥ 95%; Mw~85 kDa) was obtained from Bio21 Co., Ltd. (Chonburi, Thailand). Lactic acid (85% *w*/*v*; C_3_H_6_O_3_) was supplied by Ajax Finechem Pty Ltd. (Taren Point, NSW, Australia), and hydrogen peroxide (30% *v*/*v*; H_2_O_2_) was purchased from Merck & Co., Inc. (Darmstadt, Hessen, Germany). All remaining reagents were of analytical grade.

#### 2.1.2. Sample Preparation and γ-Irradiation Procedure

Chitosan solution was prepared by dissolving 15 g of chitosan in a beaker containing 400 mL of a 1.5% (*w*/*v*) lactic acid solution under magnetic stirring until a homogeneous solution was obtained. After that, 80 mL of the chitosan solution was transferred into four different beakers. Different amounts of H_2_O_2_ solution were added into the four beakers, and the volume was then adjusted with distilled water to obtain final H_2_O_2_ concentrations of 0%, 0.25%, 0.5%, and 1% (*w*/*v*). The samples were irradiated using a gamma 60Co irradiator at the Thailand Institute of Nuclear Technology (Public Organization) (Ongkharak, Nakorn Nayok, Thailand) with absorbed doses of 5, 10, and 20 kGy at a dose rate of 8.642 kGy/h at ambient temperature.

#### 2.1.3. Characterizations of Molecular Weight by Gel Permeation Chromatography (GPC)

Before measurement, 20 mg of dried chitosan from each condition was dissolved in 10 mL of a 2% (*w*/*v*) acetic acid aqueous solution. The molecular weight and polydispersity index of chitosan were measured using an aqueous gel permeation chromatography (GPC) system equipped with HPLC components from Shimadzu (Kyoto, Japan) and an RI detector. The GPC columns (Shodex SB-804 HQ × 1, Tokyo, Japan) were packed with polymeric polyether gels. Pullulan was used as the calibration standard. The aqueous mobile phase was operated at 0.2 M sodium acetate, with a flow rate of 0.5 mL/min and a temperature at 30 °C. A 2 mg/mL sample with a volume of 20 μL was injected.

#### 2.1.4. Viscosity by Rheometer

The size and surface charge of the chitosan solution were analyzed using a Zetasizer (Nano ZS, Malvern Instrument, Malvern, UK) with low-angle zeta potential measurement by electrophoretic light scattering. Before analysis, the chitosan solution was filtered through a 0.45 μm nylon syringe filter. Each sample was measured three times at 25 °C, and the mean and standard deviation were reported.

#### 2.1.5. In Vitro Antagonistic Assay

For the representative standard protocol assay, Gram-positive and Gram-negative pathogens, namely *Staphylococcus aureus* (Gram-positive) and *Escherichia coli* (Gram-negative), were each cultured to 1.0 × 10^8^ CFU/mL and evenly spread onto potato dextrose agar plates. Sterile paper discs were placed on the agar surface, and 10 µL of sample solution (50,000 ppm or 100,000 ppm) was applied to each disc. Plates were incubated at 37 °C and examined at 0, 12, 24, 48, and 72 h. At each time point, the diameter of the inhibition zone was measured. All assays were performed in triplicate, and the results are presented as mean ± standard deviation.

### 2.2. Application of Synergistically Degraded Chitosan in Aquafeeds to Promote Immunity, Antioxidative Status, and Disease Resistance in Nile Tilapia

#### 2.2.1. Ethics Statement

All experimental procedures involving aquatic animals strictly followed the Ethical Principles and Guidelines for the Use of Animals established by the National Research Council of Thailand for scientific purposes. Additionally, the protocol received approval from the Animal Ethics Committee at Kasetsart University, Thailand (Approval No. ACKU67-FIS-017), on 20 May 2024.

#### 2.2.2. Animal Husbandry

Healthy Nile tilapia (*Oreochromis niloticus*) juveniles (10.0 ± 2 g body weight; 4–6 cm total length) were obtained from the hatchery of the Faculty of Fisheries, Kasetsart University, Kamphaeng Saen, Nakhon Pathom Province, Thailand. Fish were acclimated in 250 L tanks at a density of 20 fish/tank. Water temperature was maintained at 28 ± 3 °C, dissolved oxygen ≥ 5 mg/L, and pH 7.0–8.0 for 30 days until the fish reached the desired experimental target size of 20 ± 5 g. Fish were fed a commercial diet containing 38% crude protein at 5% of body weight per day (split into two daily feedings), with rations adjusted weekly based on biomass sampling. Routine health monitoring including fortnightly checks for external parasites and bacterial infections was performed. Mortality was recorded daily, and any moribund fish were immediately removed. All husbandry procedures complied with institutional welfare guidelines to minimize stress and ensure optimal performance.

#### 2.2.3. Experimental Design

Both chitosan formulations, the original, non-degraded chitosan (Mw 85 kDa) and the synergistically degraded chitosan (Mw ≈ 10 kDa), were prepared by γ-irradiation at 10 kGy in the presence of 0.25% (*w*/*v*) H_2_O_2_. They were applied as dietary supplements for aquatic animals based on their antagonistic activity profiles.

A four-week feeding trial was performed using a completely randomized design (CRD) with five dietary treatments, each replicated in three 250 L fiberglass tanks. Acclimatized juvenile Nile tilapia (*Oreochromis niloticus*) (initial body weight 20.0 ± 5.0 g) were stocked at 20 fish per tank. Each treatment group consisted of four replicates, totaling 80 fish per group. The dietary treatments were as follows:

(1) C (Control): basal diet top-dressed with sterile distilled water (10% *w*/*w*).
(2) NCS-50: basal diet coated with high-molecular-weight chitosan at 0.5% *w*/*w*.
(3) RCS-0.025: basal diet coated with degraded chitosan at 0.025% *w*/*w*.
(4) RCS-0.05: basal diet coated with degraded chitosan at 0.05% *w*/*w*.
(5) RCS-0.075: basal diet coated with degraded chitosan at 0.075% *w*/*w*.

For each treatment, coating solutions were thoroughly mixed with feed pellets daily, allowed to air-dry at room temperature for 30 min, and then stored until feeding. Diets were freshly prepared each morning. All fish were fed a commercial herbivorous feed (32% crude protein; CP, Bangkok, Thailand) at 5% of body weight per day, administered in two equal rations for 4 weeks. Throughout the experiment, water temperature was maintained at 27 ± 1 °C, dissolved oxygen at ≥5 mg/L, and pH between 7.0 and 8.0. Mortality and feed intake were monitored daily, and tank biomass was measured weekly to adjust feeding rates.

At the time of sampling, fish were anesthetized using clove oil at a concentration of 50 ppm in aerated water for 3–5 min, until loss of equilibrium was observed, followed by immediate sampling.

#### 2.2.4. Growth Performance Analysis

The growth performance of all fish was evaluated at the beginning and at the end of the 4-week experiment. Before measurements, all fish were anesthetized with a 10 ppm clove oil solution for 2 min to record their weight and note any mortality. Weight gain (WG), average daily gain (ADG), feed conversion ratio (FCR), and survival rate were calculated using the following equations [14]:

WG (g/fish) = final weight − initial weight

ADG (g/day) = (final weight − initial weight)/experimental period

FCR = feed intake (g)/total weight gain (g)

Survival rate (%) = (final number of fish/initial number of fish) × 100

#### 2.2.5. Collection of Whole Blood and Serum

Blood and serum samples were collected at the end of the feeding trial (week 4). Non-anticoagulated blood was withdrawn from the caudal vein using a 1.0 mL syringe. The total RNA was immediately isolated from an aliquot of 50 µL for subsequent gene expression analysis using the Qiagen RNeasy kits (Qiagen, Hilden, Germany). The remaining blood was allowed to clot at room temperature for 1 h, after which serum was obtained by centrifugation at 7500× *g* for 10 min and stored at −20 °C until further use.

#### 2.2.6. qRT-PCR Analysis of Immune-Related Genes

At the end of the experiment, in week 4, the whole blood from Section 2.2.5, liver, head kidney, and intestine tissues were collected and preserved in PowerProtect DNA/RNA solution (Qiagen, Hilden, Germany) and briefly kept at −80 °C for further total RNA isolation assay using the Qiagen RNeasy kits (Qiagen, Hilden, Germany). Total RNA extraction from these tissues was conducted following the manufacturer’s protocol. The concentration and purity of the extracted RNA were assessed using a NanoDropTM spectrophotometer (Thermo Fisher Scientific, Waltham, MA, USA). To synthesize first-strand cDNA, 1 microliter of 1000 ng/μL total RNA was utilized as a template with Maxime™ RT PreMix (iNtRON Biotechnology, Seongnam, Republic of Korea). The resulting first-strand cDNA products were stored at −20 °C for subsequent gene expression analysis.

For qRT-PCR analysis, the QuantiNova SYBR Green qPCR Kit (Qiagen, Hilden, Germany) was used in an AriaMx real-time instrument (Agilent, Santa Clara, CA, USA). The qRT-PCR cycling conditions consisted in an initial denaturation step at 95 °C for 5 min, followed by 40 cycles of 95 °C for 30 s, 60 °C for 30 s, and 72 °C for 90 s, with a final extension at 72 °C for 10 min. The three housekeeping genes including *actb*, *ef1a*, and *gapdh* were used as internal controls to normalize mRNA and cDNA quantities and ensure result consistency. Relative expression of all target genes in fish tissues was determined using 2^−ΔΔCT^ analysis. All primers were validated and underwent efficacy testing prior to conducting the experiments. The sequences for all targeted genes are detailed in Table 1.

#### 2.2.7. Measurement of Oxidative Stress Marker and Antioxidative Enzyme Activity in Serum

(1) Thiobarbituric-Acid-Reactive Substance (TBARS) Assay

Serum malondialdehyde (MDA) was quantified by the TBARS method [21]. To 0.1 mL of serum, 0.2 mL thiobarbituric acid solution and 1.0 mL trichloroacetic acid were added, along with an equal volume of 0.85% (*w*/*v*) saline. The mixture was heated at 100 °C for 30 min and then cooled to room temperature before adding 2.0 mL of distilled water. After centrifugation at 3500 rpm for 10 min, the absorbance of the clear supernatant was read at 532 nm against a blank. MDA concentration was interpolated from a tetramethoxypropane standard curve and normalized to the protein content (µM/mg protein).

(2) Reduced Glutathione (GSH) Content

GSH levels were measured using a modified Jollow et al. protocol [22]. Serum (1.0 mL) was combined with 1.0 mL of 4% sulfosalicylic acid (*w*/*v*) and incubated at 4 °C for 1 h. After centrifugation at 3500 rpm and 4 °C for 20 min, the supernatant was mixed with a reagent containing 100 mM phosphate buffer (pH 7.4) and 100 mM DTNB. The formation of the yellow chromophore was monitored at 412 nm, and GSH concentration was determined from a GSH standard curve and expressed as mmol/mg protein.

(3) Nitric Oxide (NO) Determination

Serum nitrite, as a proxy for NO, was assayed via the Griess reaction, adapted from Sun et al. [23]. Equal volumes (0.1 mL each) of serum and Griess reagent, comprising 1% sulfanilamide in 5% phosphoric acid and 0.1% *N*-naphthylethylenediamine dihydrochloride, were mixed and incubated briefly. The resulting purple azo dye’s absorbance was read at 546 nm. Nitrite concentration was calculated against a sodium nitrite standard curve and reported as µM per mg protein.

(4) Glutathione Reductase (GR) Activity

GR activity was assessed using a modified Sahreen et al. method [24]. A 0.1 mL serum aliquot was added to a mixture of 0.1 mL 0.1 M phosphate buffer (pH 7.6), 0.1 mL oxidized glutathione (1 mM), and 0.05 mL NADPH (0.1 mM). The decrease in absorbance at 340 nm, corresponding to NADPH oxidation, was recorded. Enzyme activity was calculated using an extinction coefficient of 6.22 × 10^3^ M^−1^ cm^−1^ and expressed as µM NADPH oxidized per min per mg protein.

(5) Catalase (CAT) Activity

CAT was measured by monitoring the decomposition of hydrogen peroxide, based on Maehly’s method [25]. The assay mixture comprised 0.1 mL of serum, 2.5 mL of 50 mM phosphate buffer (pH 5.0), and 0.4 mL of 5.9 mM H_2_O_2_. Absorbance decreases were recorded at 240 nm every 30 s over 2 min. A standard curve generated with known CAT concentrations allowed expression of results as units per min per mg protein.

(6) Superoxide Dismutase (SOD) Activity

SOD activity was determined by following the procedure performed by Takada et al. [26]. Serum (0.1 mL) was mixed with 1.0 mL of assay buffer containing 0.1 mM xanthine, 0.025 mM nitroblue tetrazolium, 0.1 mM EDTA, 60 mM sodium carbonate (pH 10.2), and xanthine oxidase. The reduction in nitroblue tetrazolium was monitored at 560 nm. Activity units were interpolated from an SOD standard curve and normalized to protein content (units per min per mg protein).

(7) Glutathione Peroxidase (GPx) Activity

GPx activity was assayed with a modified Mohandas et al. protocol [27]. A 1.9 mL reaction mixture—composed of 0.1 M phosphate buffer (pH 7.4), 1 mM EDTA, 1 mM sodium azide (to inhibit catalase), 1 U/mL glutathione reductase, 1 mM reduced glutathione, 0.2 mM NADPH, and 0.25 mM H_2_O_2_—was pre-equilibrated at 25 °C. The reaction was initiated by adding 0.1 mL of serum, and the decrease in absorbance at 340 nm, corresponding to NADPH oxidation, was recorded continuously for 3 min. GPx activity (mM NADPH oxidized·min^−1^·mg^−1^ protein) was calculated using the extinction coefficient for NADPH (6.22 × 10^3^ M^−1^ cm^−1^), with appropriate blank corrections and protein normalization.

(8) Glutathione-S-Transferase (GST) Activity

GST was assayed as described by Habig et al. [28]. The reaction mixture contained 1.475 mL of 0.1 M phosphate buffer (pH 6.5), 0.2 mL of reduced glutathione (1 mM), 0.025 mL of CDNB (1 mM), and 0.3 mL of serum. Conjugation of CDNB was monitored by measuring absorbance at 340 nm. Activity was calculated from a CDNB conjugate standard curve and reported as µM of conjugate formed per min per mg protein.

#### 2.2.8. Humoral Immune Response Assays

(1) Lysozyme activity

Serum lysozyme activity was determined by the rate of *Micrococcus lysodeikticus* cell lysis following Shugar (1952) [29] with minor modifications: bacterial cells (Sigma, St. Louis, MO, USA) were washed twice in 0.05 M phosphate buffer (pH 6.2) and adjusted to an OD_600_ of 0.6. Then, 180 µL of this suspension was mixed with 20 µL of serum in triplicate wells of a 96-well plate, while blanks received phosphate buffer (pH 7.4) instead of serum. Absorbance at 540 nm was measured immediately (A_0_) and after 5 min at 25 °C (A_5_), and lysozyme activity (Unit/mL) was calculated as follows:

Enzyme (U/mL) = [(ΔA540 Sample − ΔA540 Blank) × dilution factor]/(0.001) × (0.1)

(2) Total serum IgM antibody level using a direct ELISA

Serum total IgM antibody levels were quantified by a direct ELISA as previously described by [30,31] with some modifications. Flat-bottom 96-well microplates (Thermo Fisher Scientific, Vacaville, CA, USA) were first coated with carbonate–bicarbonate buffer (pH 9.6) and incubated for 2 h at room temperature. Coating solution was discarded, and 100 µL of 1:100 diluted fish serum was added to each well and left overnight at 4 °C. Plates were washed three times with PBST (PBS + 0.05% Tween-20, pH 7.4) before blocking with 100 µL VisualProtein-BlockPRO™ (Visual Protein, Energenesis Biomedical Co., Ltd., Taipei, Taiwan) for 2 h at room temperature. After a further three washes, 100 µL of HRP-conjugated rabbit anti-tilapia IgM (1:2000; GeneScript, Piscataway, NJ, USA) was applied and incubated for 2 h at room temperature. Wells were then washed six times with PBST, and 100 µL TMB One Component HRP Substrate was added for a 1 min development at room temperature in the dark. The reaction was halted by dispensing 100 µL of TMB Stop Solution per well, and absorbance was read at 450 nm on an iMark™ Microplate Reader (Bio-Rad Laboratories, Hercules, CA, USA). Wells lacking serum served as negative controls to define the assay cutoff.

(3) Bactericidal activity

Serum bactericidal activity was evaluated against live representative Gram-positive and Gram-negative pathogenic bacteria, namely *Streptococcus agalactiae* and *Edwardsiella tarda*, respectively, using serum samples collected from experimental fish, following the protocol previously described [14,30]. In each assay, 40 µL of serum was combined with 10 µL of bacterial suspension (1 × 10^5^ CFU/mL in 0.85% NaCl) in a 96-well plate, yielding a final inoculum of 1 × 10^3^ CFU per well. The mixture was incubated for 2 h at room temperature. Survivors were enumerated by plating on Tryptic Soy Agar (TSA) and counting CFUs after 24 h of incubation. Negative controls (bacteria without serum) and sterility controls (serum without bacteria) were included to define 100% survival and 100% bactericidal activity, respectively. Bactericidal activity was calculated as follows: BA (%) = [(T_0_ − T_24_)/T_0_] × 100, where T_0_ is the number of initial bacteria, and T_24_ is the number of bacteria after plating for 24 h.

#### 2.2.9. Disease Resistance and Relative Percent Survival (RPS) to *E. tarda*

Following the feeding trial, 30 fish per treatment (10 fish per replicate) were randomly selected and transferred individually into 250 L fiberglass tanks. Each fish was challenged by intraperitoneal injection of 100 µL of a *E. tarda* suspension at 1 × 10^7^ CFU/mL (equivalent to 1 × 10^6^ CFU/fish), based on a preliminary 14-day LD_50_ determination. Mortality was monitored daily for 14 days. Relative percent survival (RPS) was calculated as follows: RPS = 1 − (mortality rate of treatment fish/mortality rate of control fish) × 100 [32].

#### 2.2.10. Statistical and Data Analysis

Growth performance, immune-related gene expressions, antioxidant activity, immune responses, cumulative survival, and RPS are expressed as mean ± standard deviation (SD). Data were statistically analyzed by one-way analysis of variance (ANOVA). The Turkey’s multiple range test was used to assess significant differences among treatment groups. All statistical analyses and graphical presentations were performed using GraphPad Prism version 10.1.2 (GraphPad Software, San Diego, CA, USA). The cumulative survival analysis of fish challenged with *E. tarda* was calculated using the Kaplan–Meier method. Cumulative survival plots were generated GraphPad Prism version 10.1.2 (GraphPad Software, San Diego, CA, USA). Statistical significance between the control and treatment groups was denoted by letter superscripts at *p* < 0.05.

## 3. Results

### 3.1. Effects of γ-Radiation Dose and H_2_O_2_ Concentration on Molecular Weight and Polydispersity Index (PDI) of Chitosan

**Figure 1:**
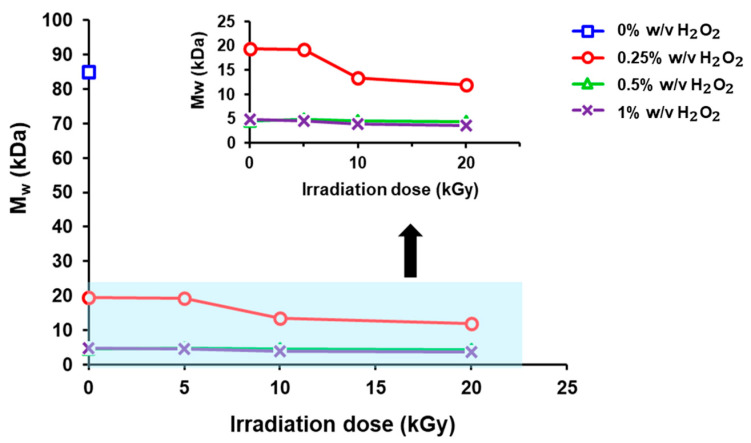
Variation in the average molecular weight (Mw) of chitosan solution as a function of γ-radiation dose and H_2_O_2_ concentration.

The initial average molecular weight of chitosan was 84.995 kDa. At a fixed H_2_O_2_ concentration of 0.25% *w*/*v*, the average molecular weight of chitosan decreased with increasing radiation dose. The molecular weights were 19.372, 19.277, 13.359, and 12.007 kDa at radiation doses of 0, 5, 10, and 20 kGy, respectively. As a result, the possibility of free radicals interacting with the chitosan polymer chain increased, leading to a decrease in average molecular weight. At H_2_O_2_ concentrations of 0.5% *w*/*v* and 1% *w*/*v*, the average molecular weight decreased significantly compared to that at 0.25% *w*/*v* under the same radiation dose. Moreover, it can be observed that the polydispersity index (PDI) after degradation with γ-radiation dose and H_2_O_2_ of the chitosan solution is closer to unity, indicating a narrow molecular weight distribution. This benefits the control of the molecular weight and stability of the chitosan solution (Figure 1 and Table 2).

### 3.2. Effects of γ-Radiation Dose and H_2_O_2_ Concentration on Viscosity of Chitosan

**Figure 2:**
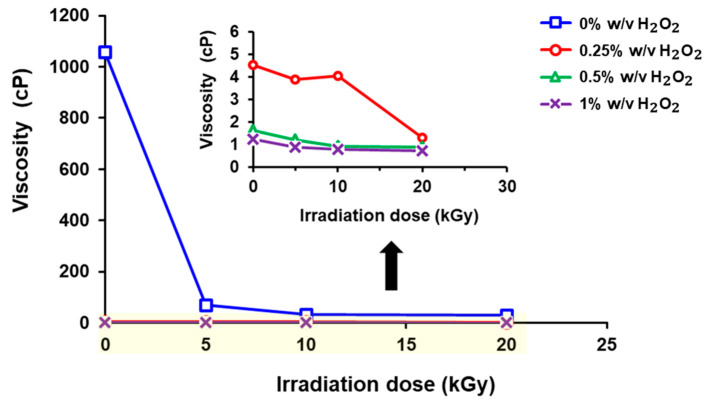
Average viscosity of chitosan solution as a function of γ-radiation dose and H_2_O_2_ concentration.

The average viscosity of chitosan prepared under various conditions is shown in Figure 2. For the non-degraded chitosan, the viscosity was very high with a value of 1057.33 ± 337.93 cP. In contrast, at γ-radiation doses of 5, 10, and 20 kGy, the viscosity of chitosan decreased significantly to 68.64 ± 1.62, 32.85 ± 5.99, and 28.38 ± 19.15 cP, respectively. Additionally, the increment of H_2_O_2_ concentration further reduced the viscosity. However, the use of H_2_O_2_ at only 0.25% *w*/*v*, in combination with γ-irradiation, resulted in a more than 200-fold reduction in viscosity compared to the initial molecular weight chitosan (non-irradiated).

### 3.3. Effects of γ-Radiation Dose and H_2_O_2_ Concentration on Size and Surface Charge of Chitosan

**Figure 3:**
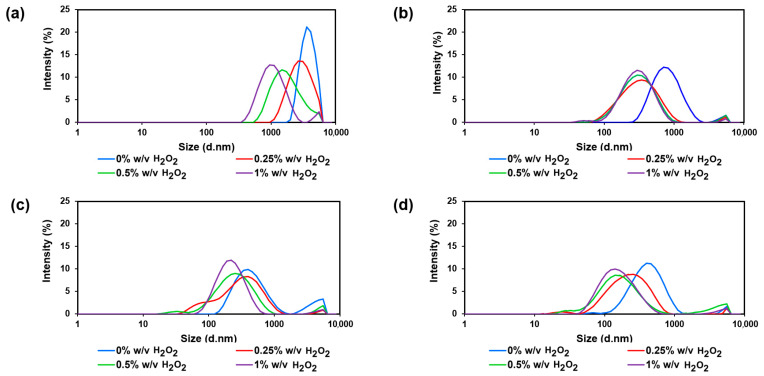
Hydrodynamic diameter distribution measured by DLS of chitosan solutions prepared at γ-radiation doses of (**a**) 0 kGy, (**b**) 5 kGy, (**c**) 10 kGy, and (**d**) 20 kGy in the presence of varying H_2_O_2_ concentrations.

The results demonstrated that higher γ-irradiation doses and increased H_2_O_2_ concentrations led to smaller average chitosan sizes, as evidenced by a leftward shift in the distribution. At a 10 kGy gamma radiation dose with H_2_O_2_ concentrations of 0%, 0.25, 0.5, and 1% (*w*/*v*), the average size of the chitosan solution decreased from 430 (0.429) nm to 224 (0.451), 215 (0.413), and 188 (0.285) nm, in that order (Figure 3 and Table 3).

The data in Table 4 present the surface charge of chitosan obtained from preparations under different γ-radiation doses and H_2_O_2_ concentrations. The surface charge of all samples in the experiment was positive due to the protonated form of –NH_3_^+^ groups in the chitosan structure. Furthermore, the surface charge was higher than ±30 mV. This is due to the repulsive force of the positive charges on the chitosan surface. The stability of degraded chitosan enhances the distribution of the chitosan solution, making it easier to apply to aquatic feed products (Table 4).

### 3.4. In Vitro Antagonistic Activity of Chitosan Synergistically Degraded by γ-Irradiation and H_2_O_2_

**Figure 4:**
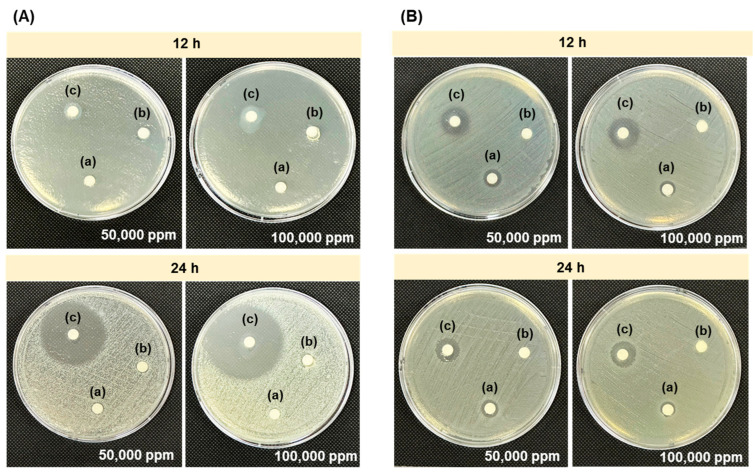
Disc diffusion assay showing inhibition zones of (**A**) *S. aureus* and (**B**) *E. coli* treated with (a) control (1.5% *w*/*v* lactic acid), (b) non-degraded chitosan, and (c) degraded chitosan at 50,000 ppm and 100,000 ppm after 12 and 24 h of incubation.

Since antimicrobial activity is one of the most important biological properties for the development of chitosan as a dietary supplement for aquatic animals, both original chitosan and chitosan synergistically degraded by gamma radiation at a dose of 10 kGy in the presence of 0.25% (*w*/*v*) H_2_O_2_ were used in this study as representative high molecular weight (Mw = 85 kDa) and low molecular weight (Mw = 13 kDa, approximately 10 kDa), respectively. The antimicrobial behavior of the samples at concentrations of 100,000 and 500,000 ppm against the growths of Gram-positive *S. aureus* and Gram-negative *E. coli* were assessed using the disc diffusion method over various incubation times. The results showed that at 12 h (Figure 4), the control (1.5% *w*/*v* lactic acid) did not show a clear zone, whereas non-degraded and degraded chitosan were observed. Specifically, degraded chitosan at 50,000 ppm and 100,000 ppm clearly showed an inhibition zone against both *S. aureus* (Figure 4A) and *E. coli* (Figure 4B) at 24 h. This can be explained by the fact that the low-molecular-weight chitosan can easily interact to inhibit the growth of both Gram-positive and Gram-negative bacteria compared to high-molecular-weight chitosan (non-degraded chitosan).

**Figure 5:**
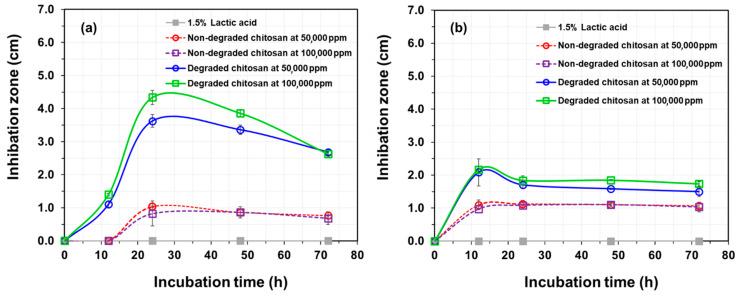
Inhibition zone diameters of (**a**) *S. aureus* and (**b**) *E. coli* bacteria treated with control (1.5% *w*/*v* lactic acid), non-degraded chitosan, and degraded chitosan at 50,000 ppm and 100,000 ppm as a function of incubation time.

Considering degraded chitosan for the treatment of *S. aureus* at 12 h (Figure 5a), the inhibition zone diameters at 50,000 ppm and 100,000 ppm were 1.11 ± 0.09 cm and 1.40 ± 0.04 cm, respectively. After 24 h, they increased to 3.63 ± 0.19 cm and 4.34 ± 0.21 cm for 50,000 ppm and 100,000 ppm, in that order. For *E. coli* treatment at 12 h (Figure 5b), the inhibition zone diameters of degraded chitosan at 50,000 ppm and 100,000 ppm were 2.08 ± 0.41 cm and 2.16 ± 0.04 cm, respectively. After 24 h, they reduced to 1.71 ± 0.11 cm and 1.84 ± 0.14 cm and remained nearly constant until 72 h. The inhibition of degraded chitosan against the growth of *S. aureus* and *E. coli* was compared at 24 h.

### 3.5. Application of Synergistically Degraded Chitosan in Aquafeeds to Promote Immunity, Antioxidative Status, and Disease Resistance in Nile Tilapia

#### 3.5.1. Growth Performance

The effects of different chitosan formulations on the growth performance of fish over a 4-week period are summarized in Table 5. All experimental groups, including control, NCS-50, RCS-0.025, RCS-0.050, and RCS-0.075, exhibited comparable weight gain (WG), average daily gain (ADG), survival rate (SR), and feed conversion ratio (FCR), with no statistically significant differences observed among the treatments (*p* > 0.05).

Specifically, the highest mean weight gain was recorded in the control group (38.50 ± 4.4 g/fish), followed by RCS-0.025 (37.50 ± 1.18 g/fish), NCS-50 (36.50 ± 2.59 g/fish), RCS-0.050 (36.17 ± 3.54 g/fish), and RCS-0.075 (35.67 ± 1.41 g/fish). Average daily gain followed a similar trend, ranging from 1.28 ± 0.36 g/day in the control group to 1.17 ± 0.27 g/day in RCS-0.050. All groups achieved a 100% survival rate throughout the experimental period. The feed conversion ratio (FCR) was slightly improved in the treated groups compared to the control, with the lowest FCR observed in the RCS-0.025 group (1.02 ± 0.02), although the differences were not statistically significant (*p* > 0.05).

#### 3.5.2. Expression of Immune-Related Genes

**Figure 6:**
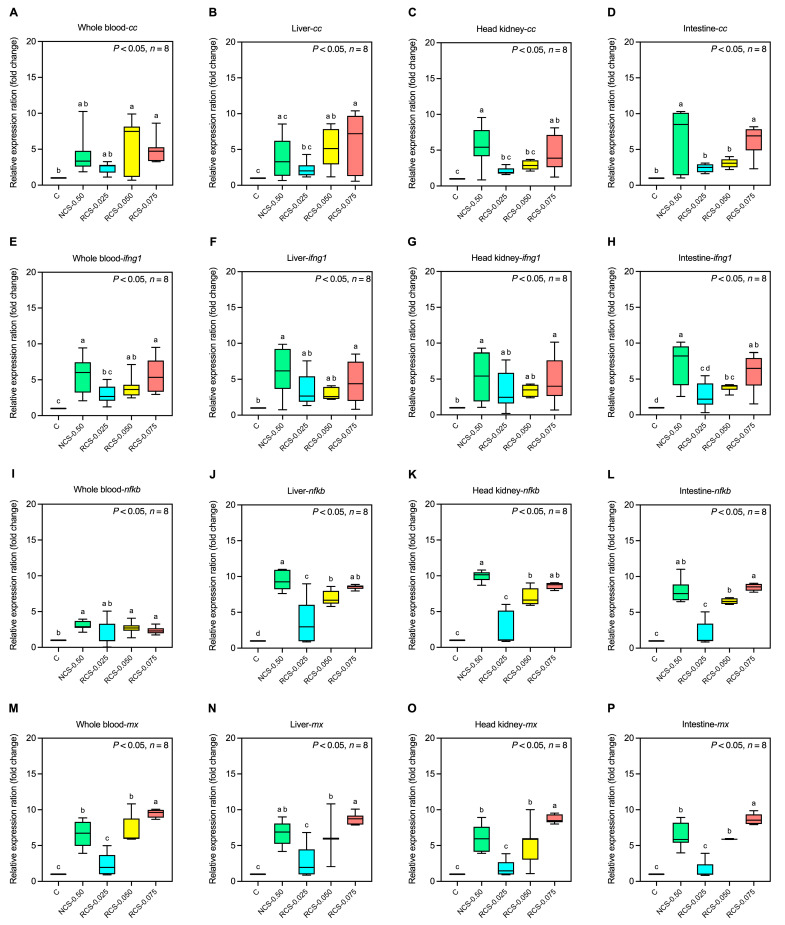

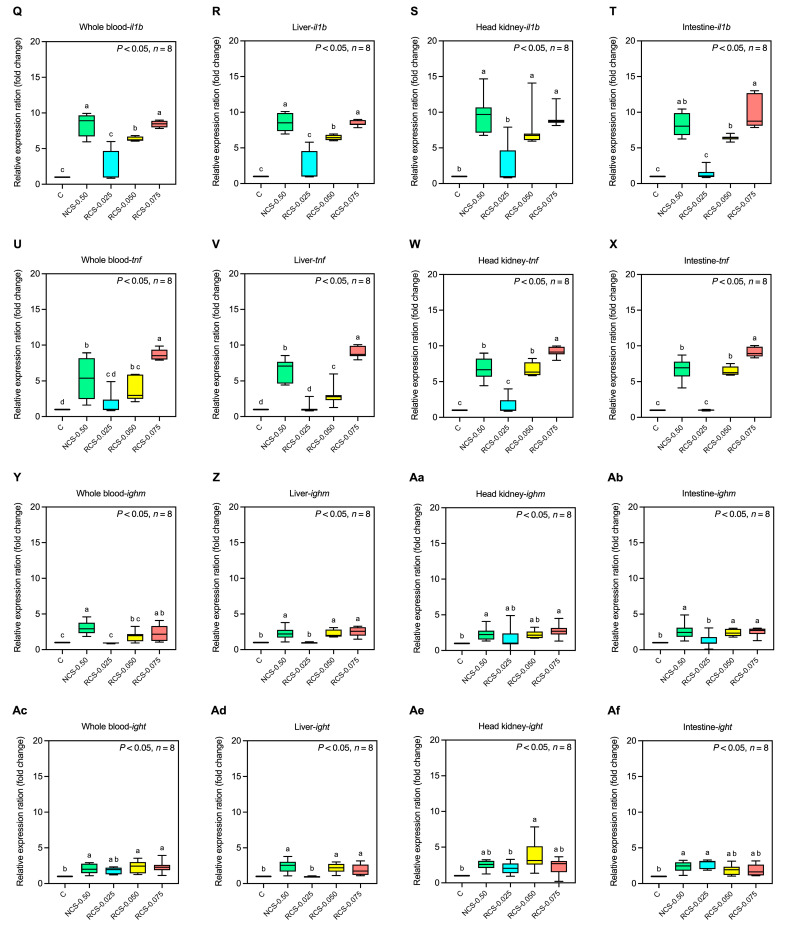
Immune-related gene expression of fish fed with different chitosan formulations over a 4-week period of whole blood, liver, head kidney, and intestine in response to *cc* (**A**–**D**), *ifng1* (**E**–**H**), *nfκb* (**I**–**L**), *mx* (**M**–**P**), *il1b* (**Q**–**T**), *tnf* (**U**–**X**), *ighm* (**Y**–**Ab**), and *ight* (**Ac**–**Af**) genes. Data are presented as mean ± SD; superscript letters within each group denote significant differences (*p* < 0.05), *n* = 8.

In the whole blood and liver, the expression of the *cc* gene was significantly higher in the RCS-0.050 and RCS-0.075 groups compared to the control group (*p* < 0.05), while the NCS-0.50 and RCS-0.025 groups showed no significant difference (*p* > 0.05) (Figure 6A,B). On the other hand, the *cc* gene expression in the head kidney and intestine was significantly higher in the NCS-0.50 and RCS-0.075 groups compared to the control group (*p* < 0.05), whereas the remaining groups showed no significant difference (*p* > 0.05) (Figure 6C,D).

The *ifng1* gene was significantly upregulated in the NCS-0.50, RCS-0.050, and RCS-0.075 groups in the whole blood and intestine (*p* < 0.05), while the RCS-0.025 group showed no significant difference when compared to the control group (*p* > 0.05) (Figure 6E–H). In the liver and head kidney, *ifng1* was upregulated only in the NCS-0.50 and RCS-0.075 groups (*p* < 0.05), while the RCS-0.025 and RCS-0.050 showed no significant difference when compared to the control group (*p* > 0.05) (Figure 6F,G).

In the liver, *nfkb* expression was significantly upregulated in all treatment groups compared to the control group (*p* < 0.05) (Figure 6J). In the whole blood, head kidney, and intestine, *nfkb* was significantly upregulated in the NCS-0.50, RCS-0.050, and RCS-0.075 groups (*p* < 0.05), while the RCS-0.025 group showed no significant difference (*p* > 0.05) (Figure 6I,K,L).

The expression of *mx*, *tnf*, and *il1b* genes was significantly upregulated in the whole blood, liver, head kidney, and intestine in the NCS-0.50, RCS-0.050, and RCS-0.075 groups (*p* < 0.05), whereas the RCS-0.025 group showed no significant increase compared to the control group (*p* > 0.05) (Figure 6M–X).

The *ighm* gene expression was significantly upregulated in the liver and intestine of the NCS-0.50, RCS-0.050, and RCS-0.075 groups (*p* < 0.05), while in the whole blood and head kidney, upregulation was observed only in the NCS-0.50 and RCS-0.075 groups (*p* < 0.05). The RCS-0.025 group showed no significant difference in any organs (*p* > 0.05) (Figure 6Y–Ab).

For the *ight* gene, the whole blood and liver in the NCS-0.50, RCS-0.050, and RCS-0.075 groups showed a significant increase in expression *(p* < 0.05), while the RCS-0.025 group showed no significant difference compared to the control group (*p* > 0.05) (Figure 6Ac–Ad). In the intestine, upregulation was observed in the NCS-0.50 and RCS-0.025 groups (*p* < 0.05) (Figure 6Af). For the head kidney, only the RCS-0.050 group showed a significant increase (*p* < 0.05), while the other groups showed no significant change when compared to the control (*p* > 0.05) (Figure 6Ae).

#### 3.5.3. Serum Oxidative Stress Marker and Antioxidative Enzyme Activity

**Figure 7:**
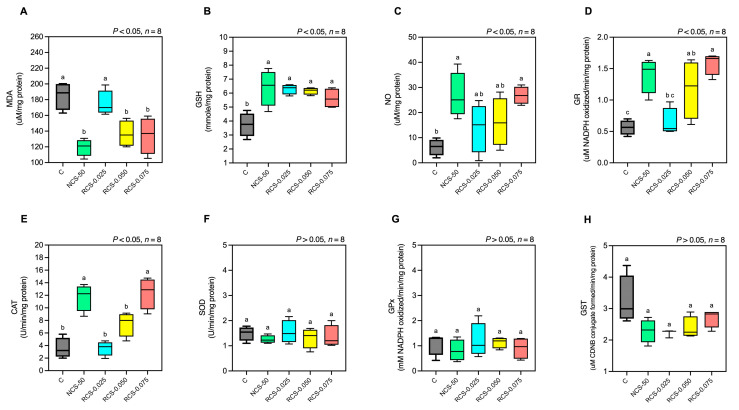
Serum oxidative stress marker and antioxidative enzyme activities of fish fed with different chitosan formulations over a 4-week period including malondialdehyde (MDA; (**A**)), reduced glutathione (GSH; (**B**)), nitric oxide (NO; (**C**)), glutathione reductase (GR; (**D**)), catalase (CAT; (**E**)), superoxide dismutase (SOD; (**F**)), glutathione peroxidase (GPx; (**G**)), and glutathione S-transferase (GST; (**H**)). Data are presented as mean ± SD; superscript letters within each group denote significant differences (*p* < 0.05), *n* = 8.

Dietary supplementation with both non-degraded and degraded chitosan markedly improved the fish’s antioxidant defenses (Figure 7). Specifically, malondialdehyde (MDA) levels, a marker of lipid peroxidation, significantly decreased from 185.0 ± 18.0 µmol/mg protein in controls to 119 ± 11, 137 ± 17, and 135 ± 23 µmol/mg of protein in the NCS-50, RCS-0.050, and RCS-0.075 groups, respectively (*p* < 0.05). Consistent with this, all chitosan-fed groups exhibited significantly higher reduced glutathione (GSH) concentrations than controls (*p* < 0.05). NO activity in the NCS-50 and RCS-0.075 groups was significantly increased compared to the control group (*p* < 0.05), while the RCS-0.025 and RCS-0.050 groups showed no significant differences compared to the control group (*p* > 0.05). GR activity in the NCS-50, RCS-0.050, and RCS-0.075 groups was significantly higher than in the control group (*p* < 0.05), whereas the RCS-0.025 group showed no significant difference, in comparison with the control group (*p* > 0.05). CAT activity in the NCS-50 and RCS-0.075 groups was significantly higher than in the control group (*p* < 0.05), while no significant differences were observed in the RCS-0.025 and RCS-0.050 groups (*p* > 0.05). SOD, GPx, and GST activities in all treatment groups showed no significant differences compared to the control group and among all treatment groups (*p* > 0.05).

#### 3.5.4. Humoral Immune Responses

(1) Lysozyme activity

**Figure 8:**
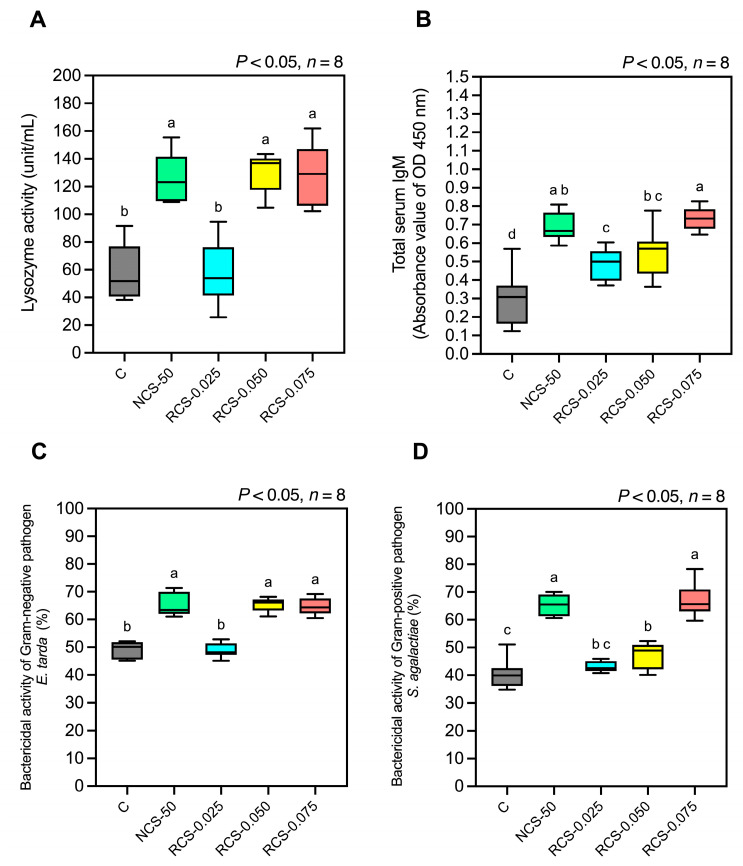
Four-week effects of experimental diets on fish immune responses: serum lysozyme activity (**A**), total serum IgM (**B**), and bactericidal activity against *E. tarda* (**C**) and *S. agalactiae* (**D**). Data are presented as mean ± SD; superscript letters within each group denote significant differences (*p* < 0.05), *n* = 8.

The lysozyme activity in the NCS-50, RCS-0.050, and RCS-0.075 groups (125.0 ± 18.03, 130.1 ± 13.52, and 129.1 ± 21.74 units/mL, respectively) was significantly higher than that in the control group (58.06 ± 20.12 units/mL) (*p* < 0.05). No significant difference was observed in the RCS-0.025 group (58.78 ± 22.67 units/mL) (*p* > 0.05) (Figure 8A).

(2) Total serum IgM antibody level

All treatment groups, including NCS-50, RCS-0.025, RCS-0.050, and RCS-0.075, showed a significant increase in total serum IgM levels, as indicated by absorbance values of 0.69 ± 0.07, 0.49± 0.08, 0.55 ± 0.13, and 0.73 ± 0.06, respectively, compared to the control group (0.29 ± 0.14) (*p* < 0.05) (Figure 8B). Furthermore, the RCS-0.025 group exhibited total serum IgM levels that were significantly lower than those in the NCS-50 and RCS-0.075 groups (*p* < 0.05).

(3) Bactericidal activity

The in vitro serum bactericidal activity against *E. tarda* (Gram-negative) and *S. agalactiae* (Gram-positive) is shown in Figure 8C,D. Against both pathogens, the NCS-50, RCS-0.050, and RCS-0.075 groups exhibited significantly higher bactericidal activity than the control and RCS-0.025 groups (*p* < 0.05), with no significant differences among NCS-50, RCS-0.050, and RCS-0.075. However, specifically for *S. agalactiae*, the RCS-0.050 group showed that the bactericidal activity was significantly lower than that of NCS-50 and RCS-0.075 (*p* < 0.05).

#### 3.5.5. Disease Resistance and Relative Percent Survival (RPS) to *E. tarda*

**Figure 9:**
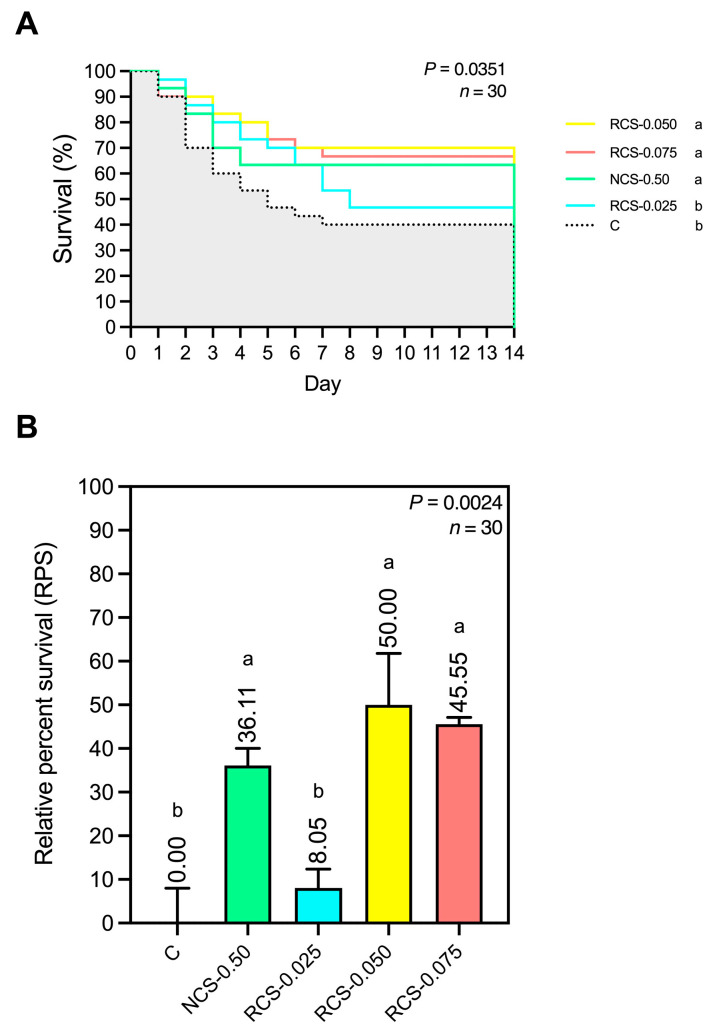
Percent survival (**A**) and relative percent survival (RPS) (**B**) of fish following intraperitoneal challenge with *E. tarda* over a 14-day period. Data are presented as mean ± SD; superscript letters within each group denote significant differences (*p* < 0.05), *n* = 30.

The assessment of fish survival following challenge with *E. tarda* revealed statistically significant differences among treatment groups (*p* = 0.0351), as illustrated in Figure 9A. Fish treated with NCS-0.50, RCS-0.050, and RCS-0.075 exhibited significantly higher survival rates than those in the control and RCS-0.025 groups. Notably, the RCS-0.050 group showed the highest survival rate, followed by the RCS-0.075 and NCS-0.50 groups, while the control and RCS-0.025 groups demonstrated the lowest survival rate. In addition, the RPS values shown in Figure 9B also indicated statistically significant differences among groups (*p* = 0.0024). The RCS-0.050, RCS-0.075, and NCS-0.50 groups exhibited RPS values of 50.00%, 45.55%, and 36.11%, respectively, which were significantly higher than those of the control and RCS-0.025 groups.

## 4. Discussion

Under the relentless barrage of γ-radiation, the aqueous chitosan solution becomes a crucible of free radicals: water molecules rupture into hydroxyl (•OH) and hydrogen radicals, and the addition of 0.25–1.0% H_2_O_2_ amplifies this oxidative maelstrom through Fenton-like reactions that spawn even more •OH. Each radical, like a microscopic scalpel, targets the C–H bonds adjacent to the 1 → 4 glycosidic linkages of the chitosan backbone, cleaving long polymer chains into shorter oligomers. As radiation dose increases from 5 to 20 kGy, the average molecular weight plummets from ~85 kDa to as low as 3.6 kDa, and the polydispersity index contracts from 2.50 to near 1.55, yielding a remarkably uniform population of fragments [33].

Freed from their entangled macroscale form, these oligomers impart dramatically lower viscosity—down from over 1000 cP to mere tens of centipoise—transforming the once-gummy solution into one that pours almost like water. Concurrently, these shorter chains collapse into nanometer-sized assemblies: dynamic light scattering reveals mean diameters shrinking from micrometer-scale clumps (≈2429 nm) to sub-200 nm nanoparticles. Each nanoparticle surface bristles with protonated –NH_3_^+^ groups, generating zeta potentials above +30 mV; the resulting electrostatic repulsion prevents aggregation, ensuring a stable colloid ideal for uniform feed coating.

When deployed against bacterial pathogens, these nanoscale chitosan fragments demonstrate superior antimicrobial prowess. Their small size and enhanced solubility allow deeper penetration through cell walls, while their polycationic surfaces bind fiercely to negatively charged components—teichoic acids in *S. aureus* and lipopolysaccharides in *E. coli.* Within 12 h, inhibition zones of up to 1.4 cm against *S. aureus* and 2.16 cm against *E. coli* emerge; by 24 h, *S. aureus* zones expand dramatically (~4.3 cm), outpacing the slower-growing zones (~1.8 cm) in Gram-negative bacteria, whose outer membrane offers partial protection. In contrast, high-molecular-weight chitosan—viscous, poorly soluble, and broadly dispersed—yields only modest inhibitory effects, underscoring how precise degradation converts a bulky biopolymer into an agile, uniform, and potent antimicrobial agent perfectly suited for aquaculture feed applications.

Extensive prior studies corroborate the dramatic physicochemical and antimicrobial transformations that were observed. For example, Shrivastava et al. (2018) [33] demonstrated that γ-irradiation alone could reduce chitosan’s Mw from ~100 kDa down to ~20 kDa at 20 kGy, but when combined with just 0.1% H_2_O_2_, Mw fell below 10 kDa—very similar to our drop to 3.6–4.8 kDa at 0.5–1.0% H_2_O_2_ under 20 kGy. Vikhoreva & Gal’braikh (1997) [34] showed that such chain scission reduces solution viscosity by over two orders of magnitude, from roughly 1200 cP to under 15 cP at comparable shear rates—again mirroring our 1057 cP → 28 cP change.

On the colloidal front, Savitri et al. (2014) [35] reported that degraded chitosan fragments below 200 nm with zeta potentials above +35 mV remained stable for weeks, resisting aggregation—consistent with our nanoparticle sizes of 148–224 nm and zeta potentials of +33–+39 mV. Finally, numerous studies have documented that low-molecular-weight (low-Mw) chitosan exhibits two- to threefold greater antibacterial activity than its high-Mw counterpart. For example, Qin et al. (2006) [36] reported significantly larger inhibition zones of low-Mw chitosan against *S. aureus*, while Jeon et al. (2001) [37] demonstrated enhanced activity of degraded chitosan against Escherichia coli, attributing the effect to improved membrane permeability and increased interaction with bacterial surfaces. Together, these data from the literature reinforce that γ-irradiation in concert with H_2_O_2_ reliably produces low-Mw, low-viscosity, colloidally stable chitosan nanoparticles with markedly enhanced antimicrobial potency [38].

This study demonstrates that both high-molecular-weight chitosan (NCS-0.50) and synergistically gamma-degraded low-molecular-weight chitosan (RCS) positively influence fish health, although their effects vary depending on the molecular size and dosage applied. Among the low-Mw chitosan treatments, 0.050% (RCS-0.050) and 0.075% (RCS-0.075) emerged as the most effective in enhancing multiple physiological and immunological parameters. These responses were comparable to those observed with the high-molecular-weight chitosan at 0.5% (NCS-0.50), despite the fact that the RCS treatments were administered at approximately one-tenth the concentration. This finding suggests that gamma-degraded low-Mw chitosan may exhibit enhanced bioactivity and efficacy at significantly lower dosages, highlighting its potential as a cost-effective and efficient immunostimulant in aquaculture feed formulations.

Despite the absence of statistically significant differences in growth performance parameters such as weight gain (WG), average daily gain (ADG), and survival rate among treatments, the consistently high values observed suggest the favorable biological safety of both high- and low-molecular-weight chitosan supplementation. These positive outcomes are likely due to the enhanced solubility and bioavailability of low-molecular-weight (Mw) chitosan at optimal doses, such as 0.050% and 0.075%. The smaller polymer size of degraded chitosan facilitates improved absorption through the intestinal lining, allowing for more effective interaction with immune and epithelial cells [39,40].

The immune gene expression profiles further highlight the immunostimulatory potential of chitosan. Dietary supplementation with NCS-0.50, RCS-0.050, and RCS-0.075 resulted in the upregulation of key pro-inflammatory and antiviral genes, including *cc*, *ifng1*, *nfκb*, *mx*, *tnf*, and *il1b*, across multiple tissues such as the whole blood, liver, head kidney, and intestine. Notably, RCS-0.050 induced significant expression in most of these immune-related genes but exhibited a more balanced, tissue-specific activation pattern compared to the broader and possibly more generalized activation observed with NCS-0.50 and RCS-0.075. This suggests that RCS-0.050 may better fine-tune the immune response, promoting effective defense mechanisms while minimizing the risk of excessive inflammation or immune fatigue [12]. The likely mechanisms involve the interaction of chitosan with pattern recognition receptors (PRRs), such as Toll-like receptors (TLR2 and TLR4), leading to activation of downstream signaling pathways including NF-κB, MAPKs, and IRFs [41,42]. These cascades regulate the transcription of cytokines and antiviral effectors like interferon-γ (*ifng1*) and myxovirus resistance protein (*mx*), enhancing both innate and adaptive immune responses. Furthermore, the upregulation of *il1b* and *tnf* reflects the early activation of pro-inflammatory pathways that are essential for pathogen clearance [43,44,45]. The absence of significant immune gene induction in the RCS-0.025 group reinforces the importance of dose optimization to achieve a meaningful immunological response.

Oxidative stress biomarkers demonstrated that NCS-0.50, RCS-0.050, and RCS-0.075 significantly reduced malondialdehyde (MDA) levels, indicating a decrease in lipid peroxidation and oxidative tissue damage. Concurrently, elevated levels of reduced glutathione (GSH) and increased glutathione reductase (GR) activity in these groups suggest an enhanced cellular redox balance and detoxification capacity. Interestingly, RCS-0.050 did not significantly increase catalase (CAT) or nitric oxide (NO) activity, in contrast to NCS-0.50 and RCS-0.075, which showed broader activation of these antioxidant markers. This may imply that RCS-0.050 induces a more regulated and tissue-specific antioxidant response, avoiding excessive or non-targeted activation of oxidative stress pathways [45]. These antioxidant enhancements are consistent with the well-documented free radical scavenging properties of chitosan, which are further amplified when the molecular weight is reduced [12,40]. Low-molecular-weight chitosan possesses higher solubility and improved cellular permeability, allowing it to more effectively interact with intracellular targets and modulate oxidative defense systems [45,46]. The observed redox improvements likely involve upregulation of phase II detoxification enzymes and the glutathione-dependent antioxidant system, contributing to overall cellular homeostasis and protection against oxidative damage [45].

Serum lysozyme activity, total IgM levels, and bactericidal activity were significantly elevated in the NCS-0.50, RCS-0.050, and RCS-0.075 groups, indicating a strong stimulation of nonspecific humoral immune responses. Among the low-molecular-weight formulations, RCS-0.050 and RCS-0.075 provided comparable enhancements to NCS-0.50, with RCS-0.050 showing slightly lower bactericidal activity against *Streptococcus agalactiae* relative to the high-Mw chitosan. These findings suggest that appropriately dosed degraded chitosan can match or even surpass the immunostimulatory efficacy of high-Mw forms in enhancing key components of innate humoral defense [45]. The observed immunopotentiation likely reflects chitosan’s ability to enhance phagocyte function and stimulate the production of opsonins such as IgM, which play a critical role in pathogen neutralization. Lysozyme activity, a well-established biomarker for innate immunity, is indicative of macrophage activation and enhanced bacteriolytic potential. The upregulation of these humoral parameters in the RCS-0.050 and RCS-0.075 groups supports the hypothesis that molecular size optimization enhances chitosan’s bioavailability and interaction with immune effector cells. In contrast, the relatively weaker immune responses observed in the RCS-0.025 group across all markers underscore the importance of threshold dosing to achieve effective immunomodulation [12,43,45].

Following challenge with *E. tarda*, fish in the RCS-0.050 group exhibited the highest survival rate and relative percent survival (RPS), significantly surpassing both the control and low-dose RCS-0.025 groups. This pronounced protective effect is likely the result of synergistic enhancements across multiple immune parameters, including both innate (e.g., lysozyme activity, bactericidal capacity) and adaptive (e.g., IgM production) responses, as well as strengthened antioxidant defenses. The integration of these mechanisms likely contributed to more effective pathogen clearance and reduced tissue damage under infectious stress. While NCS-0.50 and RCS-0.075 also conferred measurable protection, the superior survival outcomes observed in the RCS-0.050 group underscore the importance of dose optimization for low-molecular-weight chitosan. The results support the hypothesis that properly dosed degraded chitosan not only enhances immunocompetence but also provides tangible disease resistance benefits, positioning RCS-0.050 and RCS-0.075 as promising immunonutrient candidates for functional feed applications in aquaculture.

An important outcome of this study is the demonstration that low-Mw chitosan (RCS) can achieve equivalent or superior biological efficacy at lower inclusion rates compared to high-Mw chitosan (NCS). For example, the optimal RCS-0.050 diet (0.05%) provided similar or better effects than the 0.50% NCS dose, effectively reducing the required chitosan amount by tenfold. From a scientific standpoint, the enhanced bioactivity of low-Mw chitosan stems from its greater solubility, lower viscosity, and increased interaction with biological membranes and mucosal surfaces, enabling more efficient cellular uptake and immune modulation. These physicochemical advantages allow smaller doses to elicit significant physiological effects. From a practical perspective, this reduces feed formulation costs, minimizes potential interference with feed texture, and aligns with sustainable aquaculture practices by lowering input usage while maintaining high fish health performance.

## 5. Conclusions

Taken together, the findings suggest that both high- and low-Mw chitosan can enhance fish health, but the efficacy is highly dose- and Mw-dependent. The RCS-0.050 and RCS-0.075 formulations consistently provided optimal outcomes across growth, immune responses, antioxidative status, and disease resistance. These benefits likely arise from their superior solubility, bioavailability, and immunomodulatory capacity. Thus, degraded chitosan at the right dose represents a promising strategy for functional feed development in aquaculture. Further mechanistic studies are warranted to clarify the molecular pathways involved.

## Figures and Tables

**Table 1:** Primers used for gene expression analysis in this study.

Genes	Gene Group	Primer Names	Nucleotide Sequences (5′→3′)	T_m_ (°C)	Reference
*CC chemokines*	Immune-related gene	*On_cc*	F: ACAGAGCCGATCTTGGGTTACTTG R: TGAAGGAGAGGCGGTGGATGTTAT	60	[15]
*Interferon gamma 1*		*On_ifng1*	F: CAGCAGAGATGAACTTGA R: CACTAGGAAATACGGGTTT	60	[15]
*Nuclear factor-kappa B*		*On_nfkb*	F: AACGACGGTGATGACAACGAC R: AAATTCAGGCTCCACACTGACC	60	[16]
*Myxovirus resistance*		*On_mx*	F: ACCCTTGAGCTGGTGAATCA R: ATCCTGAGTGAATGCGGTCA	60	[17]
*Interleukin 1 beta*		*On_il1b*	F: GTGCTGAGCACAGAATTCCAGGAT R: GAAGAACCAAGCTCCTCTTTTGGC	60	[15]
*Tumor necrosis factor*		*On_tnf*	F: CTGTAGTCACCTCCATTA R: TACTTGTTGTTGCTTCTG	60	[15]
*Immunoglobulin M heavy chain*		*On_ighm*	F: GCAAGTAACCCAGTCACTAAAGTC R: AAGGTTCCCTCAAAGGCTCAAT	60	[18]
*Immunoglobulin T heavy chain*		*On_ight*	F: CAACAGTGGCAGTTCACATCCT R: ACGTTGGTGCCTGTAACATAAC	60	[18]
*Actin beta*	References/housekeeping gene	*On_actb*	F: ACAGGATGCAGAAGGAGATCACAG R: GTACTCCTGCTTGCTGATCCACAT	60	[19]
*Elongation factor-1-alpha*		*On_ef1a*	F: GGACACGGAAAGGATTGACAG R: GTTCGTTATCGGAATTAACCAGAC	60	[20]
*Glyceraldehyde-3-phosphate dehydrogenase*		*On_gapdh*	F: GATAATGGCAAACTTGTCGTCG R: ACATTGGAGCATCGGGTGAG	60	[20]

**Table 2:** Molecular weight (Mw) and polydispersity index (PDI) of chitosan after degradation with different γ-radiation doses and H_2_O_2_ concentrations.

Samples	H_2_O_2_ (% *w*/*v*)	Radiation Dose (kGy)	Average Molecular Weight, M_W_ (kDa)	PDI ($M_{w}/M_{n}$)
Chitosan (DD > 95%)	0	0	84.995	2.504
Degraded chitosan	0.25	0	19.372	2.530
		5	19.277	2.520
		10	13.359	2.426
		20	12.007	2.503
	0.5	0	4.571	1.553
		5	4.858	1.569
		10	4.594	1.553
		20	4.341	1.549
	1.0	0	4.813	1.621
		5	4.570	1.596
		10	3.975	1.547
		20	3.600	1.764

**Table 3:** The size and polydispersity index of chitosan prepared at different radiation doses and H_2_O_2_ concentrations.

H_2_O_2_ (% *w*/*v*)	Average Size (nm)			
	0 kGy	5 kGy	10 kGy	20 kGy
0	2429 (0.261)	701 (0.277)	430 (0.429)	370 (0.355)
0.25	2197 (0.367)	272 (0.352)	224 (0.451)	207 (0.492)
0.5	1227 (0.456)	278 (0.341)	215 (0.413)	148 (0.533)
1	922 (0.391)	266 (0.264)	188 (0.285)	137 (0.335)

**Table 4:** Surface charge of chitosan prepared at different γ-radiation doses and H_2_O_2_ concentrations.

H_2_O_2_ (% *w*/*v*)	Zeta Potential (mV)			
	0 kGy	5 kGy	10 kGy	20 kGy
0	+58.8	+42.7	+40.7	+39.5
0.25	+48.4	+42.7	+37.6	+34.2
0.5	+48.8	+38.0	+35.1	+35.4
1	+39.5	+36.5	+33.5	+33.4

**Table 5:** Growth performance of fish fed diets containing different chitosan formulations over a 4-week period.

Growth Parameters	Control	NCS-50	RCS-0.025	RCS-0.050	RCS-0.075
Weight Gain; WG (g/fish)	38.50 ± 4.4 ^a^	36.50 ± 2.59 ^a^	37.50 ± 1.18 ^a^	36.17 ± 3.54 ^a^	35.67 ± 1.41 ^a^
Average Daily Gain; ADG (g/day)	1.28 ± 0.36 ^a^	1.22 ± 0.46 ^a^	1.25 ± 0.39 ^a^	1.17 ± 0.27 ^a^	1.19 ± 0.27 ^a^
Survival Rate; SR (%)	100 ^a^	100 ^a^	100 ^a^	100 ^a^	100 ^a^
Feed Conversion Ratio; FCR	1.16 ± 0.04 ^a^	1.08 ± 0.01 ^a^	1.02 ± 0.02 ^a^	1.06 ± 0.13 ^a^	1.05 ± 0.15 ^a^

Data are presented as mean ± SD; letter superscripts within each row denote significant differences (*p* < 0.05), *n* = 60.

## Data Availability

The original contributions presented in this study are included in the article. Further inquiries can be directed to the corresponding authors.
